# An investigation of whether emotion regulation mediates the relationship between attachment insecurity and suicidal ideation and behaviour

**DOI:** 10.1002/cpp.2735

**Published:** 2022-03-29

**Authors:** Holly Turton, Katherine Berry, Adam Danquah, Jessica Green, Daniel Pratt

**Affiliations:** ^1^ Division of Psychology and Mental Health, School of Health Sciences, Faculty of Biology, Medicine and Health University of Manchester Manchester UK; ^2^ Greater Manchester Mental Health NHS Foundation Trust Manchester UK; ^3^ Manchester Academic Health Sciences Centre (MAHSC) Manchester UK

**Keywords:** adult attachment, affect regulation, betrayal trauma, emotion regulation, mediation, suicide

## Abstract

**Objective:**

The aim of this research was to examine relationships between attachment insecurity and suicidal ideation and behaviour. Secondary aims were to explore the mediating role of emotion dysregulation and the moderating role of betrayal trauma in explaining hypothesised relationships.

**Method:**

Sixty‐five participants with experience of suicidal ideation completed questionnaire measures assessing attachment security, suicide ideation, emotion regulation, betrayal trauma, depressive symptoms and hopelessness.

**Results:**

A direct relationship was found between avoidant attachment and suicide ideation after controlling for age and gender. Multiple suicide attempters had higher anxious attachment. Anxious and avoidant attachment, suicide ideation and betrayal trauma were associated with emotion dysregulation. The relationship between attachment insecurity and suicide ideation was not mediated by emotion dysregulation. In the mediation model, only anxious attachment remained a significant predictor of emotion regulation and there was no significant effect of emotion regulation nor betrayal trauma, on suicide ideation.

**Conclusion:**

Suicidal individuals may benefit from therapeutic intervention that explores attachment‐related difficulties and therapies such as dialectical behavioural therapy, which support skills in emotional regulation. Future longitudinal research should identify other important mediators of the association between attachment and suicidality to develop more targeted psychological interventions for suicidality.

**Key Practitioner Message**

- Avoidant attachment is associated with suicide ideation.
- Multiple suicide attempters have more anxious attachment.
- Emotional regulation and experiences of trauma in close relationships are associated with suicidal ideation.
- Therapists should explore the role of attachment when working with people with suicidal ideation and behaviours.

## 1 INTRODUCTION

More than 700,000 people die by suicide every year, making it a major global public health problem (World Health Organization, 2021). The act of suicide is often preceded by suicide ideation and attempts. Suicide ideation can be highly distressing for the individuals concerned (Lakeman & Fitzgerald, 2008), as well as their family members (Buus et al., 2014). It is important to determine psychological variables associated with suicidal ideation and behaviour to allow more targeted psychological interventions to prevent suicide.

Attachment theory is an important framework to support our understanding of emotional well‐being and has been applied to the understanding of suicidal ideation and behaviours. According to attachment theory, individuals develop internal working models of themselves, others and the world, based on their experiences of interaction with the attachment figure (Bowlby, 1958). A so‐called secure internal working model would be developed if an individual had experienced comfort and protection from the attachment figure, while also being supported to explore the environment. Conversely, an insecure internal working model would develop as a result of a lack of response to the need for comfort or exploration. Research into adult attachment relationships (e.g., Brennan et al., 1998) has suggested that adult attachment styles could be understood across two dimensions: avoidance and anxiety. Adults could be rated as being high or low within these dimensions and a typical secure adult would have low scores on both dimensions. People high in attachment avoidance avoid relying on others in the face of distress and shut off emotional responses. People high in attachment anxiety are easily overwhelmed by distressing emotions and are hypersensitive to being rejected by others.

Adults often continue to turn to an attachment figure when distressed and securely attached individuals are more likely to seek out and benefit from this support than those who are insecurely attached (Mikulincer & Shaver, 2007). Individuals who have experienced sensitive and responsive caregivers are likely to learn over time that seeking support results in comfort and protection. Adults with a secure attachment style may feel more confident that others can provide comfort at a time of distress and help them to manage difficult emotions. Internalising positive experiences with an attachment figure can help individuals to self‐soothe and regulate their own emotions (Mikulincer & Shaver, 2007).

Unsurprisingly, attachment insecurity is significantly more likely to be found amongst individuals experiencing psychological difficulties (Mikulincer & Shaver, 2007). Previous reviews suggest that insecure attachments are a risk factor for suicidal thoughts and behaviours (Green et al., 2020; Miniati et al., 2017; Zortea et al., 2021).

Adam (1994) was the first author to propose a developmental model of suicidal behaviour and attachment. The model suggests that early attachment experiences produce a vulnerability to suicidal behaviour, mediated by internal working models of the self and the attachment figure. The effect of these internal structures is present in difficulties with emotion regulation, relationships and poor self‐worth. These difficulties can impact on ability to cope with current loss, rejection and disappointment. Adam hypothesised that this can lead to an attachment crisis, and suicidal behaviour is viewed as extreme attachment behaviour.

Adam's theory suggests that a continuum of suicidal ideation, attempts and death from suicide may be representative of a developmental continuum of attachment insecurity and a psychological continuum of internalisation (from external to internal experience). According to Adam, suicidal ideation and behaviour ranges from interpersonal responses due to threats in the attachment relationship, to more lethal action due to negative internal working models of the self and attachment figures. An alternative approach to understanding suicidal thoughts and behaviours would be to explore whether distinct psychological and contextual factors related to attachment that could distinguish between those people who experience suicidal thoughts and those who attempt suicide (Zortea et al., 2021).

Although Adam's model proposes a relationship between insecure attachment and suicidal thoughts and behaviours, it is unclear whether insecure anxious or insecure avoidant attachment are more associated with suicidal thoughts and/or behaviours. Evidence from a recent systematic review of attachment and suicidal thoughts and behaviours suggests that all forms of insecure attachment are associated with both suicidal ideation and suicide attempts (Zortea et al., 2021). However, it is still important to examine the effects of different insecure attachment orientations across the spectrum of suicidality, as different types of mediators and moderators may help explain how specific types of insecure attachment confer vulnerability (Green et al., 2020; Zortea et al., 2021).

Protective factors are present within Adam's model, which can alter the pathway and lead individuals to more resilience or more vulnerability. Adam also proposed that the external world influences severity of suicidal behaviour through the interpersonal responses of others. A recent systematic review of studies investigating psychological and social factors in the attachment and suicide relationship, found substantial heterogeneity with regards to theoretical approaches and psychological factors investigated. However, the authors concluded that Adam's model provided a helpful framework to conceptualise disparate findings, with evidence that attachment‐related concepts such as maladaptive self‐schemas and more interpersonal difficulties may be important mediators in the attachment and suicide relationship (Green et al., 2020).

In Adam's (1994) model, emotion regulation was described as a key factor in understanding vulnerability or resilience to attachment crisis, which could ultimately result in suicidal ideation and behaviour. Emotion regulation has been conceptualised as awareness, acceptance and understanding of emotions, as well as an ability to control impulsive behaviours and use appropriate strategies when experiencing negative emotions (Gratz & Roemer, 2004). It is considered an important part of the attachment system and empirical research indicates there is an association between attachment security and emotion regulation (Mortazavizadeh & Forstmeier, 2018). In this respect, early interactions with caregivers are thought to guide individuals in managing difficult emotions (Shaver & Mikulincer, 2007).

Emotion regulation has also has been linked to suicidal ideation and behaviour. Positive associations have been found between dysregulation of emotion and suicide ideation (Martin et al., 2017; Neacsiu et al., 2018). Those who have a history of suicide attempts have reported more difficulties with emotion regulation than non‐attempters (Gómez‐Expósito et al., 2016; Neacsiu et al., 2018), and a positive relationship has been found between levels of emotion dysregulation and number of suicide attempts (Anestis et al., 2014).

Previous research has explored the role of emotion dysregulation as a mediator between attachment security and psychological wellbeing. For example, emotion dysregulation is a mediator in the relationship between attachment and symptoms of depression (Malik et al., 2015) and between attachment and mental health problems (Mortazavizadeh & Forstmeier, 2018). If emotion dysregulation plays a role in the relationship between attachment and mental health, it may also be an important factor in the relationship between attachment and suicidal ideation and behaviour. Despite the potential importance of the concept of emotional dysregulation to understanding the association between insecure attachment and suicidality, the recent systematic review of studies investigating psychological and social factors in the relationship between attachment and suicide described above, failed to find any studies focused on emotional dysregulation (Green et al., 2020).

Experiences of trauma, particularly betrayal trauma, may also have an impact on the relationship between attachment insecurity, emotion dysregulation and suicidal ideation and behaviour but have been similarly been neglected in the literature (Green et al., 2020). Betrayal trauma theory (Freyd, 1996) suggests that trauma perpetrated by an individual close to the victim has more of an impact on wellbeing. The individual has to depend on the perpetrator, so betrayal awareness may be suppressed in order to maintain the relationship, which can result in psychological difficulties. Betrayal trauma has been associated with attachment insecurity (Owen et al., 2012) emotion regulation (Goldsmith et al., 2013) and psychological wellbeing (Freyd et al., 2005). Considered within the context of Adam's model (1994), betrayal trauma could be viewed as an adverse early attachment experience which predisposes individuals to attachment insecurity, or a precipitating factor increasing vulnerability to risk of suicidal ideation and behaviour.

Difficulties with emotion regulation are higher in those who have experienced high betrayal trauma, in comparison with low betrayal trauma (Ehring & Quack, 2010). As emotion regulation is thought to develop through interactions with caregivers who are able to support and model regulation of emotion, it is unsurprising that this is difficult in the context of trauma as perpetrated by an attachment figure. If an individual with an insecure attachment style experiences high betrayal trauma, they might experience more difficulties in emotion regulation, than those with an insecure attachment style that have not experienced high betrayal trauma. More difficulties with emotion regulation may then result in suicidal ideation and behaviour for some individuals. When considering emotion regulation as a mediator between attachment insecurity and suicidal ideation and behaviour, betrayal trauma may increase the impact of the mediator, therefore acting as a moderator of the mediator.

The current study aimed to examine the relationship between attachment and suicidal ideation and behaviour, and consider the roles of emotion regulation and betrayal trauma within this relationship that are potentially important concepts within Adam's model but have been previously neglected within the literature. The following hypotheses were made:

**1.** Anxious attachment and avoidant attachment will be positively associated with suicide ideation, when adjusting for age, gender, depression and hopelessness.
**2a.** Difficulties with emotion regulation will mediate the relationship between attachment insecurity and suicide ideation.
**2b.** High betrayal trauma will moderate the effect of the mediator.
**3.** Those with a history of single or multiple suicide attempts will report more difficulties with emotion regulation and attachment insecurity, than those without a history of suicide attempts. Further, those with a history of multiple attempts will report more difficulties than those with a history of a single suicide attempt.

We did not make any a priori hypotheses about the roles of different types of attachment in either suicidal ideation or attempts given the lack of clear evidence from existing models and research. Similarly, the concepts of emotional regulation and betrayal trauma were hypothesised to be important to understanding the potential influence of both types of insecure attachment.

## 2 METHOD

### 2.1 Participants

Eligible participants were required, when asked by the researcher, to respond ‘yes’ to the question ‘have you had any thoughts of killing yourself in the past 12 months?’ Further inclusion criteria included being aged 18 years or above, having the capacity to provide informed consent, having sufficient English language ability and willing to provide the name of a responsible clinician who could be contacted in response to any risk issues. To ensure all self‐reported ratings were attributable to functional, rather than physical, causes, participants were excluded from the study if they had a primary organic disorder as reported by a responsible clinician according to the participants' clinical records. Use of substances resulting in intoxication at the time of interview also resulted in exclusion from the study.

### 2.2 Procedure

This study was approved by the Greater Manchester West NHS Research Ethics Committee (Ref #17/NW/0194). Participants were recruited through two National Health Service (NHS) Trusts within the North West of England. NHS clinicians shared information about the study with clients accessing secondary care mental health services and acute mental health inpatient wards. If clients were interested in taking part, clinicians sought consent for the researchers to contact them. Self‐referrals could also be made, using the contact details provided by clinicians or as included on the posters displayed at NHS sites, voluntary organisations and in public spaces. Following referral, researchers arranged an appointment to assess inclusion and exclusion criteria. If clients were deemed eligible and wished to take part, they were supported through the consent procedure. Participants were given the option to complete the questionnaires independently (with the researcher present) or with support from the researcher.

### 2.3 Measures

#### 2.3.1 Suicide ideation

The Beck Scale for Suicide Ideation (BSSI; Beck et al., 1979) is a 21‐item self‐report measure, which assesses the severity of suicide ideation over the past week. Typical items would include “I have no desire/a weak desire/a moderate to strong desire to kill myself.” The first 19 were summed to produce a total score from zero to 38. The last two items assess the number of and intent of previous suicide attempts and were not included in the ideation total score.

#### 2.3.2 Attachment

The Experiences in Close Relationships Scale–Revised (ECR‐R; Fraley et al., 2000) is a 36‐item self‐report measure, which assesses adult attachment security across two subscales; anxious attachment and avoidant attachment. A typical item for anxious attachment would be “I often worry that my partner will not want to stay with me” and a typical item for avoidant attachment would be “I am nervous when partners get too close to me.” Each item has seven response options, rated on a scale from one (strongly disagree) to seven (strongly agree). Higher scores on each subscale indicate higher attachment insecurity.

#### 2.3.3 Emotion regulation

The Difficulties in Emotion Regulation Scale (DERS; Gratz & Roemer, 2004) is a 36‐item self‐report measure which assesses difficulties with emotion regulation across with higher scores indicating more difficulties with emotion regulation. Typical items include “I have no idea how I am feeling” and “When I'm upset, I feel out of control.” The DERS can be used to derive a global emotional dysregulation score but also six subscale scores; 1. Nonacceptance of emotional responses (Non‐acceptance), 2. Difficulty engaging in Goal‐directed behaviour (Goals), 3. Impulse control difficulties (Impulse), 4. Lack of emotional awareness (Awareness), 5. Limited access to emotion regulation strategies (Strategies), and 6. Lack of emotional clarity (Clarity). We did not make a priori hypotheses regarding the individual subscales but present analyses with the subscales for exploratory purposes.

#### 2.3.4 Betrayal trauma

The Brief Betrayal Trauma Survey (BBTS; Goldberg & Freyd, 2006) is a 12‐item self‐report measure, which assesses traumatic events, distinguishing between events involving mistreatment from someone close to the victim, mistreatment from someone not so close to the victim and non‐interpersonal events. Typical items include “You were deliberately attacked that severely by someone with whom you were very close.” For each item there are three responses; “never,” “1 or 2 times” and “more than that.” Participants respond to each item twice, to indicate the extent of their exposure to trauma before and after the age of 18. Higher scores indicate more exposure to trauma. Within this study, only scores for high betrayal trauma items (items number 3, 5, 6, 8 and 10) were used in the analysis.

#### 2.3.5 Depression

The Patient Health Questionnaire (PHQ‐9; Kroenke et al., 2001) is a 9‐item self‐report measure, which assesses symptoms of depression, on a scale of zero (not at all) to three (nearly every day). Item 9 from this measure enquires about self‐harm and was therefore removed from the analysis.

#### 2.3.6 Hopelessness

The Beck Hopelessness Scale (BHS; Beck et al., 1974) is a 20‐item self‐report measure, which assesses hopelessness from zero to 20. Typical items include “My future seems dark to me” and “I look forward to the future with hope and enthusiasm.”

Measures of internal consistency (i.e., Cronbach's alphas) for each of the above questionnaires are presented in Table 2.

### 2.4 Statistical analysis

To define sample size, we assumed a medium to large effect size of 0.40 based on previous studies investigating associations between attachment styles and suicide ideation or behaviours (e.g., Zeyrek et al., 2009). As a result, with an alpha set at 0.05 and power at 0.80, a minimum number of 50 participants would need to be recruited to detect statistically significant correlations between pairs of measures.

Missing data were explored using the Missing Values Analysis in IBM SPSS Statistics (version 23). For the PHQ‐9, there were no missing data. For all other scales, Little's chi square statistic was non‐significant, indicating that data was missing completely at random. Expectation maximisation, a method of single imputation, was therefore used to replace a small amount of missing data (<2%). Descriptive statistics were calculated for all variables, as well as partial correlations controlling for age and gender. All variables were examined for outliers and non‐normal distribution. When parametric assumptions were not met, a bias corrected bootstrapping procedure was used, with 1000 replications, and 95% bootstrap confidence intervals were reported. Between‐group comparisons were made for all measures, depending upon participants' histories of suicide attempt(s). Distribution of data was assessed within groups. When parametric assumptions were not met, Kruskal‐Wallis tests were carried out as a non‐parametric alternative, as the F‐statistic reported by ANOVA cannot be bootstrapped in SPSS (Field, 2014). Moderated mediation analyses were conducted using Hayes (2017) PROCESS (version 3.3) model 7 for SPSS. Age, gender, symptoms of depression and hopelessness were considered for inclusion as covariates. One thousand bootstrapped samples were used to produce a sampling distribution and compute the confidence interval for the indirect effect.

## 3 RESULTS

### 3.1 Sample characteristics

Sixty‐seven participants completed the questionnaire measures. Two participants were excluded from the final sample as they later reported last experiencing suicidal thoughts over 1 year ago, and were therefore not eligible for the study. There was a final sample of 65 participants. As shown in Table 1, the majority of participants identified themselves as female (69%), white British (83%) and not in a current relationship/single (68%). Almost a third of participants were either unable to work (29%) or a student (29%) with a quarter in employment (26%). Mood disorders (57%) and anxiety disorders (40%) were the most commonly self‐reported mental health diagnoses.

**TABLE 1:** Sociodemographic characteristics

Sociodemographic variables	Total *N* = 65 *N* (%)
Gender	
Male	20 (30.8)
Female	45 (69.2)
Ethnicity	
White British	54 (83.1)
White other	4 (6.2)
Asian	3 (4.5)
Mixed White and Black Caribbean	2 (3.1)
Other	2 (3.1)
Education, highest level	
None	4 (6.2)
GCSEs or equivalent	12 (18.5)
A levels or equivalent	26 (40.0)
Undergraduate degree	9 (13.8)
Postgraduate degree	6 (12.3)
Other	8 (12.3)
Relationship status	
Single	44 (67.7)
In a relationship	8 (12.3)
Cohabiting	5 (7.7)
Married	8 (12.3)
Employment status	
Unemployed	9 (13.8)
Unable to work (due to disability, mental health, and sickness)	19 (29.2)
Employed	17 (26.2)
Student	19 (29.2)
Retired	1 (1.5)
Self‐reported psychiatric diagnoses^a^	
None/not stated	11 (16.9)
Anxiety disorder	24 (39.6)
Mood disorder	37 (56.9)
Personality disorder	14 (21.5)
Psychotic disorder	10 (15.4)
Other	4 (6.2)
Self‐reported disability^a^	
None	41 (63.1)
Physical	10 (15.4)
Learning disability/difficulty	4 (6.2)
Autism spectrum condition	4 (6.2)
Mental health	11 (16.9)
Other	1 (1.5)

^a^ Participants could report more than one diagnosis/disability therefore the total % may exceed 100.

Descriptive statistics, reliability and normality test results for the questionnaire measures are displayed in Table 2. Forty‐five (69.2%) participants reported having experienced thoughts of suicide within the past month. Forty‐eight participants (73.8%) reported a lifetime history of suicide attempts, of whom 29 (60.4%) reported multiple suicide attempts.

**TABLE 2:** Descriptive statistics, normality and reliability tests for all questionnaire measures

	Mean (SD)	Range	Kolmogorov–Smirnov test		Cronbach's alpha
			Statistic	Sig. Level	α
Suicide ideation	14.23 (9.87)	0–35	.133	.006	.943
Anxious attachment^b^	4.21 (1.42)	1.06–6.61	.078	.200	.934
Avoidant attachment^b^	3.80 (1.44)	1.11–6.67	.076	.200	.935
Emotion dysregulation	120.88 (26.65)	68–166	.076	.200	.941
Non‐acceptance	20.22 (7.37)	6–30	.173	.001	.917
Goals	19.37 (4.67)	5–25	.144	.002	.841
Impulsivity	18.98 (6.65)	6–30	.112	.041	.910
Awareness	18.83 (5.46)	7–29	.116	.030	.785
Strategy	28.11 (7.51)	11–40	.098	.200	.877
Clarity	15.37 (4.66)	5–25	.104	.081	.846
High betrayal trauma	4.68 (4.09)	0–17	.126	.012	.757
Depression^a^	14.29 (6.63)	0–24	.135	.005	.893
Hopelessness	12.09 (6.49)	0–20	.145	.002	.940

^a^ PHQ‐9 item 9 enquires about self‐harm and was removed from the analysis.

^b^ *n* = 64 due to missing questionnaire data that could not be reliability imputed.

### 3.2 Correlational analyses

Pearson's product moment correlations were conducted between all study variables, controlling for age and gender (Table 3). A significant positive correlation was found between suicide ideation and avoidant attachment consistent with hypothesis one (*r* = .364, *p* = .004), but no significant correlation was found between suicide ideation and anxious attachment. There was a significant positive correlation between overall emotion dysregulation and suicide ideation (*r* = .327, *p* = .010). Suicide ideation was only positively correlated with three of the emotion dysregulation subscales; difficulties engaging in goal directed behaviour, impulsivity and limited access to emotion regulation strategies. There was a significant moderate correlation between anxious attachment and overall emotion regulation (*r* = .544, *p* < .001), as well as all emotion regulation subscales. A significant positive correlation was found between avoidant attachment and emotion dysregulation (*r* = .368, *p* = .003). Avoidant attachment was also associated with three subscales of the DERS; non‐acceptance of emotions, lack of emotional awareness and lack of emotional clarity. Betrayal trauma did not correlate significantly with suicide ideation or anxious attachment but weakly correlated with emotion dysregulation and avoidant attachment. Significant positive correlations were found between suicide ideation, depression and hopelessness. Since hopelessness was found to be strongly correlated to suicide ideation (*r* = .709, *p* < .001), it was not included as a covariate within the mediation analysis.

**TABLE 3:** Partial correlations controlling for age and gender

Variables	1	2	3	4	5	6	7	8	9	10	11	12
1. Suicide ideation	‐	‐	‐	‐	‐	‐	‐	‐	‐	‐	‐	‐
2. Anxious attachment	.222 [−.053, .481]	‐	‐	‐	‐	‐	‐	‐	‐	‐	‐	‐
3. Avoidant attachment	.364** [.114, .594]	.358** [.141, .576]	‐	‐	‐	‐	‐	‐	‐	‐	‐	‐
4. DERS Total score	.327* [.056, .602]	.544** [.375, .691]	.368** [.125, .594]	‐	‐	‐	‐	‐	‐	‐	‐	‐
5. DERS non‐acceptance	.185 [−.072, .452]	.427** [.222, .597]	.471** [.264, .664]	.663** [.485, .809]	‐	‐	‐	‐	‐	‐	‐	‐
6. DERS goals	.278 * [.016, .549]	.323* [.087, .566]	.052 [−.210, .397]	.686** [.569, .791]	.159 [−.073, .426]	‐	‐	‐	‐	‐	‐	‐
7. DERS impulsivity	.255 * [.023, .506]	.358** [.170, .543]	.215 [−.032, .489]	.845** [.772, .904]	.427** [.196, .645]	.628** [.476, .751]	‐	‐	‐	‐	‐	‐
8. DERS awareness	.102 [−.177, .390]	.269* [.028, .506]	.339** [.106, .565]	.501** [.272, .722]	.359** [.096, .589]	.044 [−.218, .371]	.273 * [.030, .532]	‐	‐	‐	‐	‐
9. DERS strategy	.350 ** [.095, .583]	.445** [.224, .622]	.180 [−.086, .437]	.788** [.669, .873]	.354** [.086, .587]	.690** [.564, .793]	.649 ** [.470, .786]	.089 [−.189, .393]	‐	‐	‐	‐
10. DERS clarity	.206 [−.064, .500]	.508** [.329, .654]	.292* [.042, .541]	.793** [.718, .865]	.432** [.214, .633]	.488** [.273, .686]	.607** [.428, .771]	.500** [.275, .712]	.495** [.298, .674]	‐	‐	‐
11. High betrayal trauma	.188 [−.082, .455]	.209 [−.076, .460]	.299* [.021, .539]	.290* [.033, .503]	.333** [.123, .547]	.078 [−.181, .312]	.230 [−.020, .447]	.183 [−.076, .399]	.203 [−.050, .439]	.165 [−.094, .391]	‐	‐
12. Depression^a^	.395** [.185, .583]	.254* [.033, .457]	.224 [−.023, .458]	.487** [.286, .652]	.304* [.088, .487]	.331** [.116, .526]	.428** [.267, .577]	.209 [−.048, .452]	.376** [.152, .588]	.445** [.245, .620]	.180 [−.076, .417]	‐
13. Hopelessness	.709** [.595, .816]	.273* [.018, .501]	.316* [.100, .517]	.422** [.194, .627]	.241 [.000, .472]	.374** [.138, .623]	.257* [.038, .466]	.188 [−.037, .439]	.440** [.213, .659]	.304* [.079, .534]	.184 [−.088, .421]	.546** [.382, .691]

*Note*: BCa bootstrap 95% CIs reported in square brackets.

^a^ This result is following removal of item 9 which enquires about thoughts of self‐harm. Correlations were run with and without this item and it was not found to impact results.

* *p* < .05.

** *p* < .01.

### 3.3 Moderated mediation analyses

Moderated mediation analyses were carried out based on Hayes (2017) Model 7. Attachment was entered as the independent variable, suicide ideation as the dependent variable and emotion dysregulation as the mediator. High betrayal trauma was entered as a potential moderator of the mediator. Age, gender and depression (without PHQ‐9 item 9) were entered as covariates in these analyses. The covariate depression had a significant positive effect on suicide ideation.

**FIGURE 1:**
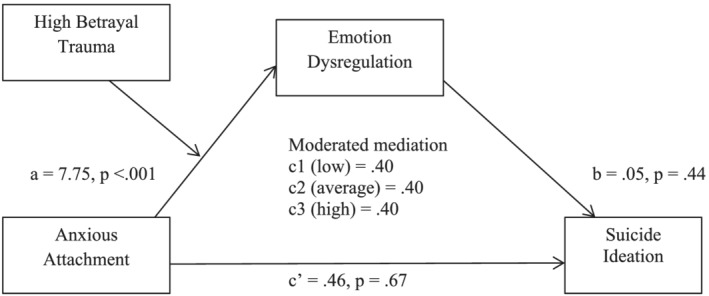
Moderated mediation model, with anxious attachment as the independent variable

In the first analysis (see Figure 1) anxious attachment was entered as the independent variable. There was a significant positive effect of anxious attachment on the mediating variable, emotion dysregulation, *b* = 7.75, *t*(57) = 4.32, *p* < .001. However, there was no significant effect of high betrayal trauma on emotion dysregulation, *b* = .91, *t*(57) = 1.23, *p* = .22, and no significant moderation effect, *b* = −.00, *t*(57) = −.00, *p* = .99. There was no significant effect of emotion dysregulation on suicide ideation. Contrary to hypothesis two, there was no significant direct or indirect effect of anxious attachment on suicide ideation and the moderated mediation model was not significant. The overall model was significant, *F*(5, 58) = 3.75, *p* < .001, *R* ^2^ = .22.

**FIGURE 2:**
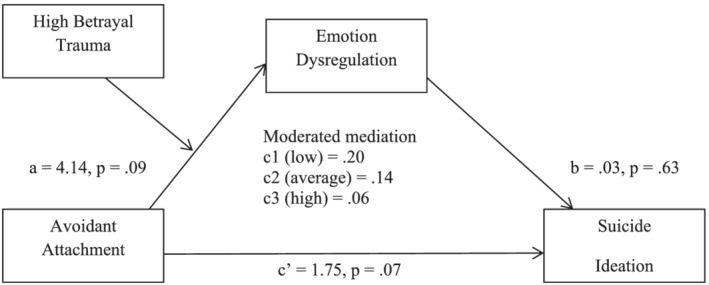
Moderated mediation model, with avoidant attachment as the independent variable

When avoidant attachment was entered as the independent variable (see Figure 2), there was no significant effect of avoidant attachment on emotion dysregulation and no significant interaction (*b* = −.48, *t*(57) = −.83, *p* = .41). There was no significant direct or indirect effect of avoidant attachment on suicide ideation and the moderated mediation model was not significant. The overall model was significant, *F*(5, 58) = 6.26, *p* < .001, *R* ^2^ = .27.

### 3.4 Comparison of means

Mean comparisons were carried out, in order to explore differences in variables between those who had reported a history of suicide attempt once, more than once or no such history. The means, standard deviations, test statistics and significance levels are displayed in Table 4. Significant differences were found between groups for suicide ideation, anxious attachment, total emotion dysregulation (but not for any of the emotion regulation subscales) depression and hopelessness. For these variables, pairwise comparisons were conducted to explore the differences between groups. Calculated effect sizes and adjusted *p* values for the pairwise comparisons are included in Table 4.

**TABLE 4:** Pairwise comparisons

	Number of previous suicide attempts			Kruskal–Wallis		Pairwise comparisons (*p* value)		
	None (*N* = 17)	Single (*N* = 19)	Multiple (*N* = 29)	*H* statistic	Sig.	None‐single	None‐multiple	Single‐multiple
Suicide ideation	11.06 (8.15)	9.58 (6.40)	19.15 (10.61)	11.10	.004	.072	**−.361** *	**−.436** **
Anxious attachment^a^	3.32 (1.16)	4.38 (1.18)	4.64 (1.50)	10.37	.006	−.372	**−.474** **	−.114
Avoidant attachment^a^	3.20 (1.20)	3.71 (1.51)	4.23 (1.42)	5.38	.068	‐	‐	‐
Emotion dysregulation	112.65 (23.81)	113.79 (20.71)	130.34 (29.25)	7.93	.019	−.040	**−.358** *	−.324
Non‐acceptance	18.94 (7.33)	19.42 (7.07)	21.48 (7.63)	2.03	.363	‐	‐	‐
Goals	18.06 (5.47)	18.42 (4.35)	20.76 (4.11)	5.35	.069	‐	‐	‐
Impulsivity	16.94 (5.55)	17.63 (6.21)	21.07 (7.11)	4.73	.094	‐	‐	‐
Awareness	18.35 (5.72)	17.11 (4.34)	20.24 (5.75)	4.08	.130	‐	‐	‐
Strategy	27.06 (6.83)	25.79 (6.92)	30.24 (7.91)	5.34	.069	‐	‐	‐
Clarity	13.29 (3.82)	15.42 (4.90)	16.55 (4.67)	4.89	.087	‐	‐	‐
High betrayal	3.45 (3.17)	3.75 (3.76)	6.01 (4.46)	5.33	.070	‐	‐	‐
Depression^b^	12.12 (7.45)	12.37 (6.78)	16.83 (5.22)	7.14	.028	−.020	−.332	−.317
Hopelessness	9.84 (5.42)	9.87 (6.21)	14.85 (6.36)	11.06	.004	.005	**−.398** *	**−.408** *

^a^ *N* = 28 for multiple attempt group due to missing data.

^b^ PHQ9 item 9 enquires about self‐harm and was removed from the analysis.

* *p* < .05.

** *p* < .01.

Those with multiple suicide attempts reported significantly higher attachment anxiety (*r* = −.474, adj. *p* = .004) and emotion dysregulation (*r* = −.358, adj. *p* = .045) in comparison with those without a history of attempts. There was no significant difference in attachment anxiety or emotion dysregulation between single and multiple attempters, or between single and non‐attempters. Multiple attempters reported significantly higher suicide ideation and hopelessness, in comparison to single attempters and non‐attempters. However, there was no significant difference in these variables between non‐attempters and single attempters. For the depression measure, no significant differences were found between groups.

## 4 DISCUSSION

The aim of the current study was to explore the relationship between attachment and suicidal ideation and attempts, and consider the role of emotion regulation and betrayal trauma within this relationship. The hypothesis that attachment insecurity would be positively associated with suicide ideation was partially supported by the results of this study. There was a positive correlation between avoidant attachment and suicide ideation but not between anxious attachment and suicide ideation. This suggests that those who are uncomfortable depending on, and becoming emotionally close to others, may also be more likely to think about suicide. Avoidant attachment style is associated with “deactivation” of the attachment system. In order to avoid distress in response to an unavailable caregiver, individuals will become more self‐reliant and suppress their needs and vulnerability (Mikulincer & Shaver, 2007). Considering Adam's (1994) model, suicide could be viewed as the ultimate deactivation of the attachment system. Avoidant individuals may think about suicide as a way of rejecting others and life itself (Miniati et al., 2017). Thinking about suicide may also serve as a distraction from difficult emotions. Some individuals have described suicide ideation as a coping mechanism that helps them to regain control (Lakeman & Fitzgerald, 2008) and avoidant attachment has been associated with a need for control (Mikulincer & Shaver, 2007).

However, the lack of association between anxious attachment and suicide ideation is unexpected. Previous research has suggested that both anxious and avoidant attachment are associated with suicide ideation (Palitsky et al., 2013). However, Grunebaum et al. (2010) found that avoidant attachment, but not anxious attachment, was predictive of suicide ideation at a three‐month follow up, but neither were associated with suicide ideation at a one‐year follow up. These mixed findings might suggest that other psychological factors associated with attachment insecurity such as relationship difficulties or poor self‐worth may contribute to increased suicide ideation, rather than a specific category of attachment‐related experiences.

The hypothesis that emotion regulation would mediate the relationship between attachment and suicide ideation and that this would be moderated by betrayal trauma, was not supported by the results of this study. Although a significant relationship between emotion regulation and suicide ideation was found, there was no significant effect of emotion regulation on suicide ideation in the mediation model. This indicates that other factors may play a part in the relationship between emotion regulation and suicide ideation. This is supported by other research that has indicated that although the two variables are correlated, emotion dysregulation does not remain a significant predictor of suicide ideation when assessed alongside other variables such as psychopathological state (Mallorquí‐Bagué et al., 2018), PTSD symptom severity (Martin et al., 2017), insomnia symptoms, manic/depressive symptoms (Palagini, Cipollone, Masci, et al., 2019) and chronobiological rhythm alterations (Palagini, Cipollone, Moretto, et al., 2019).

Anxious and avoidant attachment were found to be associated with emotion regulation, but in the mediation analysis only anxious attachment remained significant. This suggests that those with a strong desire for closeness to others and fears of abandonment are more likely to have difficulties regulating emotions. As those with an avoidant attachment style are likely to suppress emotion, they may also be less likely to self‐report difficulties with emotion regulation. Previous research has indicated under‐reporting of psychological difficulties by individuals with avoidant attachment patterns, when compared to reporting of clinicians (Dozier & Lee, 1995).

Betrayal trauma was significantly associated with avoidant attachment and emotion dysregulation, but not with anxious attachment or suicide ideation. Only anxious attachment remained a significant predictor of emotion regulation in the mediation model. It is therefore unsurprising that betrayal trauma did not moderate this relationship, as it was not associated with the attachment style that was predictive of emotion dysregulation. Although betrayal trauma has been associated with emotion regulation and attachment, further research is required to understand the role within this relationship. A more indirect relationship may be more likely whereby emotion regulation could be associated with betrayal trauma since betrayal trauma could be more likely to result in an insecure attachment style; rather than betrayal trauma directly contributing to difficulties with emotion regulation.

These findings suggest that although there is some relationship between these variables, emotion regulation does not account for the relationship between attachment and suicide ideation. Adam (1994) suggested that “personality difficulties” may explain the link between attachment and suicide, including poor self‐worth and difficulties with relationships, as well as emotion dysregulation. Given the preliminary nature of the contradictory findings from the current study, further exploration of these difficulties is required to provide evidence to support or refute Adam's model.

The results of the study partially met the hypothesis that those with a history of suicide attempts will report more difficulties with emotion regulation and attachment insecurity. There was no significant difference between groups for avoidant attachment. Those who had attempted suicide multiple times had significantly higher anxious attachment and emotion dysregulation than those who had never attempted suicide. However, there was no significant difference between attachment or emotion regulation between non‐attempters and single attempters, or between single and multiple attempters.

The findings suggest that those with multiple suicide attempts are more anxiously attached. Anxious attachment style is associated with “hyperactivation” of the attachment system. In response to an inconsistent caregiver, individuals may intensify needs in order to demand love and attention (Mikulincer & Shaver, 2007). It could be suggested that multiple suicide attempts are a way of expressing unmet needs, as supported by Adam's theory (1994) that suicidal behaviour is an extreme attachment behaviour. However, if the individual's aim at the time of suicide attempt is to activate the attachment system and receive love and care, this contradicts the idea that the individual intended to die. Those with a history of multiple suicide attempts have described ambivalence around the decision to end their life (Bergmans et al., 2017). Research has suggested that there is an association between avoidant attachment and objective suicidal intent but this relationship was not found for anxious attachment (Levi‐Belz et al., 2013). This could indicate that when anxious individuals engage in suicidal behaviours, there is less intent to die or more ambivalence around this, than when avoidant individuals make attempts. In Adam's model, anxious attachment may therefore be placed at the more ‘external’ end of the internalisation continuum than avoidant attachment, and suicide attempts for anxiously attached individuals may more frequently be in response to interactions that threaten an attachment relationship. Further research is needed in order to evidence Adam's (1994) model, but as many studies rely on self‐reports regarding highly emotive experiences, it may be difficult to accurately determine intent to die and the role of relational difficulties at the time of the suicide attempt.

The results also suggest that those who have difficulty with emotion regulation are more likely to repeatedly attempt suicide. In line with Adam's theory (1994), emotion regulation may result in difficulties coping with current relational difficulties, which may in turn lead to more frequent attachment crises and suicidal behaviours.

These preliminary findings from the current study indicate a difference in the presentation of participants with single and multiple suicide attempts. It may be that other factors are contributing to the relationship between anxious attachment, emotion regulation and suicide attempts. In a 10 year longitudinal study exploring differences between multiple and single attempters, results indicated that multiple attempters were more likely to meet the criteria for a diagnosis of Borderline Personality Disorder (BPD) and had higher impulsivity scores (Boisseau et al., 2013).

A diagnosis of BPD has been associated with attachment insecurity, particularly anxious attachment (Agrawal et al., 2004) as well as emotion regulation and suicide ideation and behaviour (Linehan, 2015). Within Adam's (1994) model, the “personality difficulties” (emotion regulation, poor self‐worth and relationship difficulties) described as a vulnerability to suicide risk, are consistent with a diagnosis of BPD. As multiple attempters are also more likely to meet BPD criteria for diagnosis, Adam's model would suggest that in these individuals, suicide ideation and behaviour would also fall at the ‘external’ end of the psychological continuum, as suggested for anxiously attached individuals. Increasing understanding into suicide ideation and behaviour in those given a diagnosis of BPD, may help to reduce stigma around this diagnosis and advise intervention for this population.

Impulsivity is a subscale of the DERS and higher impulsivity scores would have contributed to higher overall DERS scores in this study. In the current study, no significant differences were found in impulsivity scores between those with and without a history of suicide attempts but impulsivity was significantly correlated with suicide ideation. Further exploration of subscales of emotion regulation may help to develop a more fine‐grained understanding of emotion dysregulation amongst those with suicide ideation alone, as well as single and multiple suicide attempters. In the correlational analyses within this study, impulsivity was also associated with anxious attachment. It may be that in those with anxious attachment, suicide attempts were more of an impulsive reaction to an attachment crisis.

## 5 LIMITATIONS

This study used a cross‐sectional design, so no causal inferences can be made about the relationship between variables. Self‐report data also mean responses could have been subject to recall bias. Attachment was measured using the ECR‐R, which only provides scores for anxious and avoidant attachment. The measure does not offer a score for disorganised attachment style, a fourth category of attachment described which has previously been associated with suicidal ideation and behaviours (Miniati et al., 2017).

This study explored the role of emotion regulation as an overall construct. DERS total scores were used in the mediation analysis, covering awareness and acceptance of emotions as well as how they are responded to. The DERS also provides subscale scores so that elements of emotion regulation can be analysed separately. Unfortunately, due to the sample size and number of variables within the analysis, the study would not have been sufficiently powered to investigate the mediating effect of subscales, as well as total DERS.

## 6 FUTURE RESEARCH AND CLINICAL IMPLICATIONS

Longitudinal research is required in order to make any causal inferences regarding the impact of attachment style on suicide ideation or attempts. Replication studies with larger sample sizes could more closely examine the role of other psychological variables within this relationship, including the subcomponents of emotional dysregulation. In line with Adam's (1994) model, future research into this area could look further at poor self‐worth, which was one of the difficulties (alongside emotion regulation and relationship difficulties) that may contribute to the link between attachment and suicide ideation and behaviour. The model also proposed that current loss, rejection and disappointment were potential factors that precipitate suicide ideation and behaviour, while anxiety, anger and hopelessness were considered as contributing factors.

The three‐step theory of suicide (Klonsky & May, 2015) emphasises the role of connectedness in the development of potentially lethal suicide ideation. Connectedness was described as connection to people, purpose or meaning that keeps an individual invested in living. It would be interesting to explore this factor as a mediator in the relationship between attachment and suicide ideation and attempts, as it is likely that an insecure attachment style would impact on an individual's ability to connect. Cognitive inflexibility or rigidity, is another psychological variable which has been associated with suicide (O'Connor & Nock, 2014) and attachment (Mikulincer & Shaver, 2007; Salande & Hawkins, 2017) but to the author's knowledge, has not been explored alongside both variables.

Identification of mediating factors in the relationship between attachment and suicide ideation and behaviour is important in order to guide clinical intervention. Results from this study suggest that individual patterns of relating to others are important areas to consider when working with clients at risk of suicide. Exploration of attachment experiences in initial assessments may be useful in helping to identify areas of difficulty and focus for intervention. Clinicians could help to increase awareness of relational patterns and internal working models, encouraging reflection on how they were developed. Clients could then be supported to adapt internal working models and make decisions about how to respond to future relational difficulties. In order to increase awareness and understanding of these difficulties if they arise within the therapeutic relationship, clinicians would benefit from supervision that supports exploration of process issues.

As emotion dysregulation was associated with suicide ideation and multiple suicide attempts in this study, it may be beneficial to consider strategies to increase emotion regulation skills when providing clinical intervention for individuals currently experiencing suicide ideation or those with previous attempts. Dialectical Behaviour Therapy (DBT) is a clinical intervention that includes emotion regulation skills as a key component of the programme and research has indicated that DBT reduces suicidal behaviour (Panos et al., 2014).

## 7 CONCLUSION

The current study demonstrates that emotion regulation, moderated by betrayal trauma, is not a mediator in the relationship between attachment and suicide ideation. The results do, however, provide further support for an association between attachment insecurity and suicide ideation and behaviour. Avoidant attachment was associated with suicide ideation and anxious attachment was associated with multiple suicide attempts. Therapeutic intervention that explores attachment‐related difficulties and supports individuals to adapt patterns of relating to others might be important in reducing suicide ideation and attempts.

## Data Availability

Data for this paper are available from the corresponding author upon reasonable request.
